# The Auto-Brewery Syndrome: A Perfect Metabolic “Storm” with Clinical and Forensic Implications

**DOI:** 10.3390/jcm10204637

**Published:** 2021-10-10

**Authors:** Ricardo Jorge Dinis-Oliveira

**Affiliations:** 1 TOXRUN—Toxicology Research Unit, University Institute of Health Sciences, Advanced Polytechnic and University Cooperative (CESPU), CRL, 4585-116 Gandra, Portugal; ricardinis@sapo.pt or ricardo.dinis@iucs.cespu.pt or ricardinis@med.up.pt; Tel.: +351-224-157-216; 2 Department of Public Health and Forensic Sciences and Medical Education, Faculty of Medicine, University of Porto, 4200-319 Porto, Portugal; 3 UCIBIO-Applied Molecular Biosciences Unit, REQUIMTE, Laboratory of Toxicology, Department of Biological Sciences, Faculty of Pharmacy, University of Porto, Rua de Jorge Viterbo Ferreira nº 228, 4050-313 Porto, Portugal

**Keywords:** auto-brewery syndrome, endogenous ethanol, gut microbiome, pathophysiology, diagnosis, fungi, yeast, *Saccharomyces cerevisiae*

## Abstract

Auto-brewery syndrome (ABS) is a rare, unstudied, unknown, and underreported phenomenon in modern medicine. Patients with this syndrome become inebriated and may suffer the medical and social implications of alcoholism, including arrest for inebriated driving. The pathophysiology of ABS is reportedly due to a fungal type dysbiosis of the gut that ferments some carbohydrates into ethanol and may mimic a food allergy or intolerance. This syndrome should be considered in patients with chronic obstruction or hypomotility presenting with elevated breath and blood alcohol concentrations, especially after a high carbohydrate intake. A glucose challenge test should be performed as the confirmatory test. Treatment typically includes antifungal drugs combined with changes in lifestyle and nutrition. Additional studies are particularly needed on the human microbiome to shed light on how imbalances of commensal bacteria in the gut allow yeast to colonize on a pathological level.

## 1. Introduction

Auto-brewery syndrome (ABS; also known as gut fermentation syndrome, endogenous ethanol fermentation or drunkenness disease) is a rare medical condition in which intoxicating quantities of ethanol are produced through the endogenous fermentation of carbohydrates within the digestive system, which has already been described in children and adults of both sexes [1]. The yeast *Saccharomyces cerevisiae* (i.e., brewer’s yeast), well known for its use in producing bread and alcoholic beverages, and taken by some people as a probiotic supplement, has been identified as the main causative agent for this condition, but several other microorganisms have been identified, including bacteria [2]. Indeed, it was recently shown that *Klebsiella pneumonia* can similarly ferment carbohydrates into alcohol in the gut, which can accelerate nonalcoholic fatty liver disease (NAFLD) [3]. ABS has been mainly described to occur in patients with short bowel syndrome after surgical resection, required in the context of Crohn’s disease in adults and necrotizing enterocolitis in young children [4]. The lack of a functional small intestine may predispose patients to the fermentation of malabsorbed carbohydrates and symptoms such as diarrhea, which can result in dehydration, malnutrition, weight loss, bloating, heartburn, nausea, vomiting, abdominal discomfort, fatigue, lactose intolerance, and foul-smelling stool [5,6]. A very interesting review was provided by [2].

From a forensic point of view, ABS has been reported to be the cause of endogenous ethanol production as a defense against drunk driving charges [7], but this metabolic disturbance is far from being clarified, and judges should be aware that this condition could necessitate a different judicial decision. Therefore, as a precautionary measure, physicians recommend that these patients be very careful while driving a motor vehicle. Moreover, this syndrome may have implications for programs that test urine for alcohol, including addiction treatment and transplant programs [8]. Aiming to alert clinicians, a previously unrecognized ABS was described, in which ethanol was produced through endogenous fermentation in the urinary system [8].

This article aims to fully review and discuss ABS from both clinical and forensic points of view, as these aspects are mutually dependent.

## 2. Methods

An exhaustive search was carried out in PubMed (U.S. National Library of Medicine) without a limiting period, concerning the pathophysiology, signs and symptoms, clinical history, diagnosis, treatment, causal microorganisms, and forensic aspects of ABS. Both human and nonhuman studies were included. Although case reports are known to increase the risk of bias, these were inevitably consulted, but only if the ascertainment, causality, and reporting were carefully analyzed as suggested by Murad et al. [9]. This determination was particularly based on whether studies reported strict monitoring of patients during diagnostic testing. Furthermore, retrieved journal articles, as well as books, general newspapers, and government documents, were reviewed for possible additional publications related to this topic.

## 3. Epidemiology

In 1948, Ladkin and Davies [10] reported the first case of endogenous alcohol production in a 5-year-old African boy who died after developing a perforation in the posterior abdominal wall due to excessive gas distention. Since then, several case reports have been reported in the literature involving children and adults of both sexes. The first major case series of ABS was reported in the Japanese literature in the 1970s [11], and it has been suggested that the Japanese population is particularly prone to this syndrome [1]. The syndrome is called “meitei-sho” in Japanese, which means “intragastrointestinal ethanol fermentation syndrome,” “alcohol autointoxication syndrome”, or “endogenous alcohol intoxication syndrome” [1]. It is important to remember that many Asians inherit an inactive form of low K_m_ aldehyde dehydrogenase (ALDH), which converts acetaldehyde to acetate [12]. Defective ALDH associated with higher hepatic concentrations of acetaldehyde could result in a reduction of acetaldehyde to ethanol via a reversible NAD^+^/NADH reaction catalyzed by alcohol dehydrogenase (ADH) [12]. Nevertheless, several cases from other countries involving Caucasian and Black populations have been reported [13,14,15]. Table 1 compiles the major characteristics of some reported cases related to ABS.

## 4. Etiology, Risk Factors and Pathophysiology

While ABS can occur in healthy individuals and has multifactorial causation, it is most prevalent in people with comorbid conditions, such as diabetes, obesity-related liver disease, Crohn’s disease, massive small bowel dilation, short bowel syndrome, laparotomy, gastrectomy, antibiotic therapy, immunosuppression (e.g., treatment with azathioprine) or autoimmune conditions, periodontal bacteria, and a high-carbohydrate diet, such as during diet supplementation [2,5,26,27]. In 2016, authors from the Mayo Clinic stated that ABS “…may be considered in a patient with chronic obstruction or hypomotility presenting with elevated serum ethanol levels in the setting of high carbohydrate intake” [5]. Surgical models in animals have failed to produce ABS [28,29]. Recent studies have shown that Roux-en-Y gastric bypass surgery induces a clear alteration in the structure and composition of the gut fungal and bacterial microbiota in morbidly obese patients [30]. Diabetes mellitus may also be a risk factor for yeast overgrowth and ABS. Hafez et al. [13] demonstrated significantly higher blood alcohol concentrations (BAC) in diabetic and liver cirrhosis patients than in a control group. Even though the BAC in patients with diabetes mellitus was found to be significantly higher than that of the control group, the amounts did not increase enough to affect legal proceedings [31].

**Figure 1:**
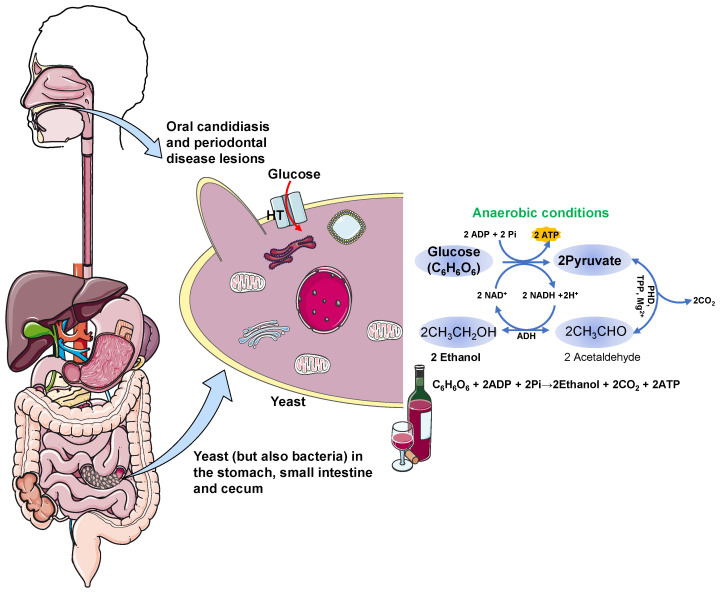
Yeast and bacterial fermentation process of the metabolism of glucose to ethanol. First, glucose is transported into the cell by the hexose transporter (HT). The process then involves the decarboxylation of pyruvic acid to acetaldehyde under anaerobic conditions, followed by the reduction of acetaldehyde to ethanol. ADH, alcohol dehydrogenase; ADP, adenosine diphosphate; ATP, adenosine triphosphate; NAD^+^, nicotinamide adenine dinucleotide; P, phosphate; PHD, pyruvate decarboxylase (an enzyme absent in humans but present in certain yeasts and bacteria); TPP, *thiamine* pyrophosphate.

The human microbiome is now recognized as an organ with a broad range of metabolic activities. In particular, the gut microbiota presents a dense and highly diverse microbial community composed of bacteria, fungi, phages, viruses, protozoa, and archaea that inhabits the gastrointestinal tract and is intimately involved in host physiology [32]. Its composition and how it affects health and disease varies according to the method of delivery at birth, being breastfed, gender, age, use of antibiotics, educational level, and the body site [33,34]. Antibiotic use, especially if it is frequent or long-term, may interfere with the gut’s natural microbiome and contribute to yeast overgrowth [35]. In particular, fungi comprise less than 2% of the total gut microorganisms, with only approximately 60 genera and 180 species [36]. An impairment of the gut microbiota, referred to as “dysbiosis” and characterized by changes in its composition and function, is commonly observed in gastrointestinal and systemic diseases [37,38,39]. *Candida* *albicans* and *Saccharomyces cerevisiae* are the main reported microorganisms involved, since they can anaerobically convert carbohydrates to endogenous ethanol and carbon dioxide (Figure 1) [40], and have been detected in intestinal secretions and stool [22]. Nevertheless, other microorganisms, such as *Candida* *glabrata*, *Candida* *intermedia*, *Candida krusei*, *Candida* *parapsilosis*, and *Candida* *kefyr*, and bacteria, such as *Enterococcus faecalis* and *Klebsiella pneumoniae*, have been implicated [41,42]. In general, *Saccharomyces cerevisiae* and *Candida albicans* are acidophilic and, as such, grow better under acidic conditions in the optimal pH range of 4 to 6 [1,43,44]. Therefore, hypoacidity of the stomach, the predigestion of carbohydrates, and the back flow of duodenal contents into the stomach or the stagnation of substrate in the intestine all provide a favorable environment. Indeed, the administration of cimetidine or antacids resulted in higher ethanol levels in the gastric juice than among the controls [45]. More recently, the in vitro ethanol production by *Candida albicans* in blood proved to be dependent on temperature, time, glucose (or carbohydrate) content, pH of the medium, and endogenous changes in the medium composition over time [46]. Therefore, microbial ethanol production, either in vitro after sample collection or in situ in corpses before autopsy, could complicate the results and mislead interpretations [47,48]. Since ethanol is mostly absorbed in the stomach and the small intestine, colonization of these intestinal segments seems to have major implications. In one case, ABS was described for the first time due to oral *Candida albicans* colonization, but mostly due to *Streptococcus* and *Klebsiella pneumoniae* bacteria from periodontal disease lesions, both demonstrating higher ethanol-producing capabilities than fungi [27].

## 5. Diagnosis

This disease can produce profound effects on everyday life through several unspecific, recurring signs and symptoms of ethanol intoxication; namely, neurological, gastrointestinal, respiratory, and psychological/psychiatric symptoms, poor productivity, and problems with employment [2,6,49]. For the diagnosis of ABS, an interprofessional approach is helpful. Since it is a very rare diagnosis and, in the past, was sometimes considered a myth, ABS is usually detected when someone fails a breathalyzer test or seeks medical help for symptoms commonly associated with ethanol intoxication, with a BAC that may be significantly higher than the legal limit (Table 1). The widespread availability of breathalyzers for purchase has facilitated the diagnosis and monitoring of these patients. ABS might be hypothesized in any patient presenting with an elevated BAC who denies ingestion, including those arrested for driving under the influence of ethanol [22]. Diagnosis should include a medical history and specific complementary diagnostic exams as described in Table 2. The hallmark for diagnosis is strict supervision and a monitoring of the patient to rule out undisclosed ethanol consumption. Moreover, even under supervision, false negative results can be obtained since previous predisposing conditions cannot be mimicked in entirety. In addition to intestinal fungal dysbiosis, signs of yeast infections in other systems (e.g., integumentary and nail beds) have also been reported [6]. Since gut dysbiosis can result in chronic excess ethanol production, this may contribute to the pathogenesis of NAFLD [3,50].

Differential diagnoses for other, more common diseases and food intolerances should be made, including D-lactic acidosis, which is a rare neurological syndrome that occurs in individuals with short bowel syndrome or following jejunoileal bypass surgery, with symptoms typically appearing after a high carbohydrate intake [53,54,55]. A previous study that compared ethanol producers and nonproducers demonstrated no significant differences in lactulose breath hydrogen [56]. In postmortem examinations, it is necessary to evaluate whether the source of the BAC is the result of putrefactive production or antemortem consumption.

## 6. Treatment and Prognosis

The random manifestation of signs and symptoms of intoxication in ABS may justify a relative obscurity of the condition and can make it difficult to seek treatment [52]. First, patients with an extremely high BAC should be treated for acute ethanol intoxication; namely, respiratory stabilization and fluid administration [57]. Second, drug therapy based on culture and sensitivity results for the identified yeast or bacteria can be implemented, with a course of one or more of the antimycotic drugs azoles (e.g., fluconazole, itraconazole), polyenes (e.g., nystatin, micafungin), and echinocandins (e.g., caspofungin) [58]. A nutritionist might be involved in the treatment and management of the disease, since an essential diet modification requiring high protein (e.g., eggs, almonds, oats, cheese, Greek yogurt, milk, broccoli, lentils, pumpkin seeds, fish, peanuts, and sprouts) and low carbohydrates (i.e., avoiding white bread, white rice, flour, pasta, and high-fructose corn juice sugars, including glucose, fructose, and dextrose) until symptoms subside is critical in ABS to decrease ethanol fermentation in the gastrointestinal tract and relapse [26]. Other lifestyle changes are recommended, such as weight control, limiting corn syrup, and eliminating ethanol intake and foods with high yeast and molds, such as coffee, peanuts, and corn. Environmental exposure to molds and yeast (e.g., grain silos, house mold) must be considered as well [18].

Single-strain *Lactobacillus acidophilus*, which has 3 billion colony-forming units or multi-strain probiotic supplements that help balance bacteria and competitively inhibit fungal growth for the recolonization and normalization of gut flora in the digestive tract, has been used in the treatment of ABS, but further studies are required [14,22]. Vandekerckhove et al. [25] first successfully treated a patient with ABS by using a fecal microbiota transplantation, in which the patient’s daughter volunteered to be the donor. This treatment was performed after the failure of more traditional therapy with a low-carbohydrate diet combined with antimycotic fluconazole or nystatin.

## 7. Forensic Aspects

Those concerned with ethanol intoxication in clinical and forensic settings must bear in mind that ABS is most likely an underdiagnosed condition. Under normal situations, and with few exceptions, the BACs reported in peripheral venous blood are vanishingly small and therefore lack clinical or forensic significance. A large study from the United Arab Emirates found by headspace gas chromatography/mass spectrometry that in 1557 participants of different nationalities, ages, and sexes, the median endogenous ethanol (i.e., physiological blood ethanol) level was 0.00113 g/L [59]. The maximum BACs reported in this study were 0.0352 and 0.0320 g/L in males and females, respectively. Other studies have revealed that the concentration of endogenous ethanol in the peripheral venous blood of healthy individuals, as well as those suffering from various metabolic disorders (e.g., diabetes, hepatitis, and cirrhosis), ranges from 0 to 0.0008 g/L [7]. A study from Saudi Arabia involving 1400 subjects belonging to different nationalities detected very small endogenously produced ethanol levels using gas chromatography and mass spectrometry [60]. The study, performed to establish gastrointestinal ethanol levels to advise on legal limits, revealed a mean endogenous BAC of 0.0014 g/L, with a maximum of 0.0153 g/L and 0.0141 g/L in males and females, respectively, all far from the cutoff point of positive ethanol levels related to forensic issues. Indeed, any endogenous ethanol generated by microbiological activity in the gut is effectively cleared when the portal venous blood passes through the liver for the first time. This is due to the abundance of hepatic ADH and the operation of Michaelis–Menten elimination kinetics with a km value of 0.05–0.10 g/L, which is efficient in metabolizing ethanol at low concentrations [61,62]. Nevertheless, these conclusions are most likely based on normal situations, as cases of ABS may not be present. A clearance rate of 0.1 g ethanol/kg body weight/h has been reported, which signifies that an adult with a body weight of 70 kg can dispose of approximately 7 g/h due to either metabolism or breath and urine excretion [7,63]. Lester [64] concluded that the “normal” BAC of humans ranges up to 0.0015 g/L. The reported reference values are the result of continuous production from acetaldehyde, which is a metabolic byproduct of pyruvate, threonine, deoxyribose-5-phosphate, phosphoethanolamine, aniline, and possibly other substrates [65]. The variability in reference values for endogenous ethanol is probably the consequence of variabilities in carbohydrate intake, starvation, aging, stress, cooling, adrenalectomy, and factors that may increase ethanol levels, such as concomitant therapy with 4-methylpyrazole and the intake of lingberry juice containing approximately 40 g of carbohydrates [65,66]. ABS was criticized by some authors [7,67] when commenting on a case of a child with short gut syndrome [20], due to the forensic implications that endogenous ethanol production may cause. Indeed, significant medicolegal issues are present in the consideration of ABS due to the potential for inappropriate use in legal defense strategies. Although rare, the BAC may exceed the legal limit in cases of ABS, and the individual may present with signs of intoxication [13]. Episodes of ABS have been reported after drinking normal, voluntary quantities of alcohol, and in such cases, it is impossible to determine the contribution to the BAC from drinking versus endogenous gut production.

In one case, the patient was kept under supervision for 24 h without any possibility of consuming alcohol, and a high BAC was obtained, providing evidence for the forensic and clinical relevance of this condition [16]. The role of ABS in the pathogenesis of sudden infant death syndrome (SIDS) has also been reported, but there is little evidence to support this argument [15,68]. Finally, although forensic experts should keep an open mind to the possibility of ABS, they should also not overestimate ABS.

## 8. Discussion and Concluding Remarks

Although ethanol is one of the most widely used and accepted recreational psychoactive substances worldwide, it has well-known negative medical and social consequences, as previously reviewed [69,70,71,72]. However, some individuals might suffer from these consequences, being misdiagnosed as ethanol abusers, without consuming any ethanol due to ABS. Indeed, this rare syndrome affects people worldwide, and very little is known about the lifestyle, health, diet, and medical history of these patients [6]. Moreover, no large-scale studies have aimed to establish diagnostic criteria or find effective treatments, although antifungals, dietary changes, and fecal microbiota transplantation could be considered for future studies. Fermentation in the gut is a normal part of the digestive process and occurs through the breakdown of food by normal bacteria in the colon. However, in people with ABS, fermentation may occur further up the digestive tract, such as in the mouth, stomach, small intestine, and cecum. Certain fungi, such as *Candida albicans* and *Saccharomyces cerevisiae*, have been found to be responsible for converting carbohydrate-rich foods into ethanol. In recent years, many cases of ABS have been reported, but researchers are far from clarifying the pathophysiology, diagnosis, and specific treatment would be interesting. Although rarely reported, recently it was suggested that ABS may represent an underdiagnosed medical condition [22].

## Figures and Tables

**Table 1:** Major findings of case reports on ABS.

Major Findings	Reference
A 38-year-old male with a BAC of 2.58 g/L the day of his traffic accident BAC of 1.60 g/L at the time of admission and 1.41 g/L, 3.22 g/L, 2.08 g/L, and 2.79 g/L after 2 h, 6 h, 12 h, and 20 h, respectively Liver function test results were high and neurological examination was normal Patient did not have a history of prior surgical procedures and did not have any gastrointestinal system diseases except for laxity in the lower esophageal sphincter and erosive gastritis	[16]
A 45-year-old obese, male, diabetic patient Treated with antibiotics for deviated nasal septum and dental procedure Reported episodes of diarrhea, vomiting, edema, seizures, hallucinations, intermittent fevers, chills, slurred speech, and loss of consciousness precipitated after meals for a duration of 14 months *Saccharomyces cerevisiae* positive in fecal contents Responded well to oral fluconazole but relapsed after a month and needed further assessment Growth of *Klebsiella pneumoniae* and *Enterococcus faecium* on gastric and small bowel contents Carbohydrate diet and intravenous administration were effective	[17]
A 13-year-old female After being restricted from access to ethanol in a rehabilitation center, she showed signs and symptoms of drunkenness	[14]
A 60-year-old male Patient and his wife both thought he had early dementia BAC of 1.70 g/L Due to a prediabetic condition, the patient cut out all sugar from his diet for several months, explaining why his episodes of drunkenness decreased dramatically Treatment with nystatin and the patient was counseled on a low-carbohydrate diet, including the avoidance of cheese and coffee, which can contain mold	[18]
A 42-year-old female Appeared highly intoxicated; reported episodes of loss of balance, slurred speech, and becoming argumentative Patient had previously broken her ribs, her nose, and split her eye/head due to loss of coordination Patient had been a social drinker in the past but then completely abstained from alcohol BAC several times above the legal limit without consumption Patient’s stool culture tested positive for *Saccharomyces cerevisiae*	
A 3-year-old female with short bowel syndrome Signs of alcohol intoxication on repeated occasions; namely, after the administration of a *Lactobacillus plantarum*-containing carbohydrate-rich fruit drink Cultures from gastric fluid showed the presence of *Candida kefyr* (also in feces) and *Saccharomyces cerevisiae*	[19]
A 13-year-old female with short gut syndrome secondary to neonatal jejunal atresia Admitted for psychiatric evaluation after repeated bouts of auto-brewery Endoscopic aspirates grew abundant *Candida* and she was temporarily treated with antifungal therapy with complete symptom resolution BAC ranged from 2.50–3.50 g/L	[20]
A 61-year-old female Uncontrolled diabetes and glycosuria with positive test for yeast *Candida glabrata* in urine Was almost rejected for liver transplant surgery, as tests showed ethanol in the urine, with negative blood results This condition was called bladder fermentation syndrome	[8]
A 24-year-old Japanese female with yeast growth due to ingested carbohydrates Slight dilatation of the duodenum and frequent backward movement of duodenal contents into the stomach Maximal BAC of 2.54 g/L A 24-year-old Japanese male with yeast growth due to ingested carbohydrates and history of appendectomy Identified factors included *Candida albicans* and *Candida krusei*, in feces The research group had previously linked endogenous alcohol fermentation to *Candida* and other yeast in inactive loops of the bowel when excess carbohydrates were present [21] A total of 39 Japanese cases (ages ranging from 1 to 75 years) were also reviewed. Most showed various gastrointestinal abnormalities resulting from abdominal surgery, such as gastrectomy, laparotomy, and cholecystectomy	[1]
A 5-year-old African male presented with gastric distension and decreased consciousness Died after rupture of the posterior gastric wall with ethanolic gastrointestinal contents despite no native medicine or beer intake for the treatment of yaws Microbiology cultures of the peritoneal contents revealed Gram-negative cocci and bacilli without any yeast	[10]
A previously active, healthy, 46-year-old male complained of having memory loss, mental changes, aggressive behavior and episodes of depression for over 6 years These changes started to occur after he received the antibiotic cephalexin for a complicated traumatic thumb injury Was arrested for presumed driving while intoxicated (BAC was 2.00 g/L), but denied alcohol ingestion *Saccharomyces cerevisiae* and *Saccharomyces boulardii* were detected in his stool and his normal stool bacterial flora The antibiotic altered his gut microbiome, allowing fungal growth in the upper small bowel and cecum Treatment with antifungal agents allowed the subsequent ingestion of carbohydrates without symptoms	[22]
A 45-year-old male with diabetes mellitus Reported episodes of stumbling and confusion, with abdominal distention, lower extremity edema, and weight gain BAC at admission was 0.65 g/L Stool studies were negative for bacterial or yeast infection, including *Saccharomyces* Fluconazole did not impact symptoms or home BAC Improved symptoms and reduced BAC with adherence to a low-carbohydrate diet and daily probiotic	[23]
A 46-year-old male who had been operated on for pyloric stenosis *Candida* was isolated as a factor in samples taken after toxification signs	[24]
A 47-year-old male who experienced intermittent episodes of feeling drunk after consecutive courses of amoxicillin–clavulanic acid and moxifloxacin for a respiratory tract infection BAC of 1.60 g/L History of Roux-en-Y gastric bypass surgery 14 years earlier Fecal culture identified *Candida glabrata* With a 100 g oral dose of glucose, BAC rose from zero to a maximum value of 0.41 g/L 4 h later Low-carbohydrate diet combined with antifungals fluconazole or nystatin failed Fecal microbiota transplantation was successful in resolving symptoms	[25]
BAC of 0.40 g/L after consuming an alcohol-free, high-carbohydrate diet Patient’s BAC was due to bacteria rather than a fungal infection because anti-fungal treatments had no effect on this parameter Strains of *Klebsiella pneumoniae* were found to be strongly associated with endogenous alcohol production	[3]
A 71-year-old male with a 50-year history of Crohn’s disease Recent long-standing obstruction/hypomotility Acute onset of dizziness and slurred speech Underwent a small bowel resection of unknown length in 1969 Hence, he began to increase his intake of sugar to gain weight Blood ethanol levels were elevated despite abstinence from alcohol for over 30 years Blood ethanol level was found to be 2.34 g/L and then 1.70 g/L and 1.25 g/L Fluid collected proximal to the stricture grew a large amount of *Candida glabrata* Changing the patient to a low-carbohydrate diet and avoiding antibiotics were sufficient to prevent a recurrence	[5]

**Table 2:** Diagnosis of ABS according to [6,22,51,52]. BAC, blood alcohol concentration; BrAC, breath alcohol concentration.

**Complete clinical history:** signs and/or symptoms of unexplained ethanol intoxication, namely neurological (e.g., seizures, slurred speech, loss of coordination leading to falls, blurred vision, fainting, memory loss), gastrointestinal (e.g., nausea, vomiting, diarrhea, generalized abdominal discomfort, irritable bowel syndrome), psychological/psychiatric (e.g., depression, bizarre behavior, somnolence, disorientation, chronic fatigue, state of mental confusion) and respiratory (e.g., runny nose, recurrent sinusitis, cough, malodorous breath), belching, xerostomia, hangovers, and episodes of a high-carbohydrate diet More likely in a patient with chronic intestinal obstruction, gastroparesis, short bowel syndrome, diabetes, or liver dysfunction, such as nonalcoholic fatty liver disease (NAFLD) or nonalcoholic steatohepatitis (NASH) **Laboratory tests:** blood cell count, metabolic panel, bacterial and fungal stool culture, fungal speciation, and antifungal sensitivity **Upper and lower endoscopy:** to collect gastrointestinal secretions from the stomach, small intestine and cecum for fungal and bacterial culture and sensitivity testing Discharge the possibility of secret drinking by monitoring ethanol intake for a period BAC and BrAC analyses may need to be repeated at multiple times of the day to detect fluctuations Drug screen analysis **Confirmatory test:** carbohydrate challenge of 200 g glucose with BAC and BrAC testing at intervals of 0, 0.5, 1, 2, 4, 8, 16, and 24 h. ABS is positive if levels are elevated during any endpoint. The test can be terminated if levels become elevated, or testing can be continued until 16 and 24 h to avoid false negatives since some fungi can take 24 h or longer to metabolize carbohydrates
